# U-shaped association between plasma sphingosine-1-phosphate levels and mortality in patients with chronic systolic heart failure: a prospective cohort study

**DOI:** 10.1186/s12944-020-01262-2

**Published:** 2020-06-04

**Authors:** Yanbo Xue, Wei Jiang, Qiong Ma, Xiqiang Wang, Pu Jia, Qiang Li, Shuping Chen, Bingxue Song, Ya Wang, Jingwen Zhang, Jing Liu, Guodong Yang, Yuyao Lin, Jing Liu, Linyan Wei, Caijuan Dong, Haiquan Li, Zhonglei Xie, Ling Bai, Aiqun Ma

**Affiliations:** 1 Department of Cardiovascular Medicine, First Affiliated Hospital of Xi’an Jiaotong University; Shaanxi Key Laboratory of Molecular Cardiology; Key Laboratory of Environment and Genes Related to Diseases, Ministry of Education, No. 277 West Yanta Road, Xi’an, 710061 Shaanxi Province People’s Republic of China; 2 Department of Pharmacy, the Second Affiliated Hospital of Air Force Medical University, No. 1 Xinsi Road, Xi’an, Shaanxi China; 3 Key Laboratory of Resource Biology and Biotechnology in Western China, College of Life Sciences, Northwest University, No. 299 Taibai Road, Xi’an, Shaanxi China; 4 Department of Epidemiology and Biostatistic, School of Public Health Xi’an Jiaotong University Health Science Center, No. 76 West Yanta Road, Xi’an, Shaanxi China; 5 The Affiliated Hospital of Qingdao University, No. 1 Jiangsu Road, Qingdao, Shandong China; 6 Department of Cardiology, the First Affiliated Hospital, Sun Yat-Sen University, No. 58 Zhongshan Road, Guangzhou, China

**Keywords:** Heart failure, Sphingosine-1-phosphate, All-cause mortality, Prognosis

## Abstract

**Background:**

The endogenous lipid molecule sphingosine-1-phosphate (S1P) has received attention in the cardiovascular field due to its significant cardioprotective effects, as revealed in animal studies. The purpose of our study was to identify the distribution characteristics of S1P in systolic heart failure patients and the prognostic value of S1P for long-term prognosis.

**Methods:**

We recruited 210 chronic systolic heart failure patients from June 2014 to December 2015. Meanwhile 54 healthy people in the same area were selected as controls. Plasma S1P was measured by liquid chromatography-tandem mass spectrometry. Patients were grouped according to the baseline S1P level quartiles, and restricted cubic spline plots described the association between S1P and all-cause death. Cox proportional hazard analysis was used to determine the relationship between category of S1P and all-cause death.

**Results:**

Compared with the control group, the plasma S1P in chronic heart failure patients demonstrated a higher mean level (1.269 μmol/L vs 1.122 μmol/L, *P* = 0.006) and a larger standard deviation (0.441 vs 0.316, *P* = 0.022). Based on multivariable Cox regression with restricted cubic spline analysis, a non-linear and U-shaped association between S1P levels and the risk of all-cause death was observed. After a follow-up period of 31.7 ± 10.3 months, the second quartile (0.967–1.192 μml/L) with largely normal S1P levels had the lowest all-cause mortality and either an increase (adjusted HR = 2.368, 95%CI 1.006–5.572, *P* = 0.048) or a decrease (adjusted HR = 0.041, 95%CI 0.002–0.808, *P* = 0.036) predicted a worse prognosis. The survival curves showed that patients in the lowest quartile and highest quartile were at a higher risk of death.

**Conclusions:**

Plasma S1P levels in systolic heart failure patients are related to the long-term all-cause mortality with a U-shaped correlation.

**Trial registration:**

*CHiCTR*, ChiCTR-ONC-14004463. *Registered 20 March 2014*.

## Introduction

Heart failure is the terminal state of a variety of cardiovascular diseases that affects the health of patients and increases the burden on society. Despite formal drug treatments, the 5-year mortality of heart failure patients remains high [1, 2]. Searches for new biomarkers with long-term prediction value and early identification of high-risk patients to reduce mortality are always important for guiding clinical treatments. Heart failure activates a variety of systemic compensatory stress responses, including stimulation of the sympathetic catecholamine system, the renin angiotensin aldosterone system and other systems. In recent years, a large number of studies have found that immune system activation and dysfunction are present during the occurrence and development of heart failure, and these immunity disorders always occur simultaneously with abnormalities of the neurohumoral endocrine system. Therefore, in-depth investigations of these pathophysiological changes call for appropriate molecules to connect the two systems together.

The circulating immunoregulatory factor sphingosine-1-phosphate (S1P), an endogenous lipid molecule, is produced by the phosphorylation of sphingosine by sphingosine kinases. S1P is an intracellular secondary messenger, and it can also enter the circulation and bind to the G protein-coupled receptor family of sphingosine-1-phosphate receptors 1–5 to play an immunomodulatory role [3, 4]. In recent years, studies have shown that S1P interacts with β-adrenergic receptors to play a role in simultaneously regulating immune system and sympathetic nervous system functions [5, 6]. Meanwhile, S1P has received attention due to its important role in cardiac ischaemia-reperfusion injury [7, 8] and ventricular remodelling inhibition [9, 10]. It is interesting that previous in vitro and in vivo studies of S1P levels and its receptor targeting activities reveal a common feature that whether the S1P level is elevated or depressed in the experimental group, the heart-protecting effect is always achieved through adjusting the S1P/S1PR axis function to the level of the normal control group. These results suggest that keeping S1P levels in the normal range may have protective effects on the heart [11, 12].

Until now, it has been unclear whether there are abnormal S1P levels in clinical patients with chronic heart failure (CHF), what its normal level is, what its clinical significance is, and whether it is related to poor prognosis. The purpose of our study was to identify the distribution characteristics of S1P levels in the blood circulation of outpatients with stable systolic heart failure and its prognostic value for long-term prognosis.

## Methods

### Patient selection

**Fig. 1:**
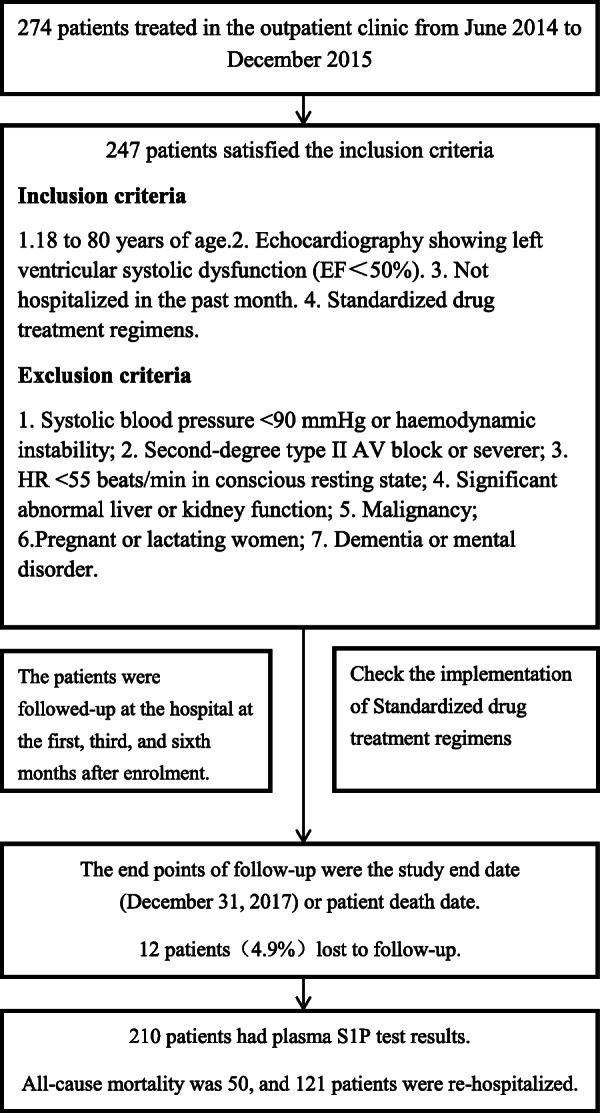
The study design and population

We recruited systolic heart failure patients treated in Jingyang Hospital and Xunyi Hospital, both located in Shaanxi Province, and who were diagnosed according to the “2014 China Heart Failure Diagnosis and Treatment Guideline” and “2012 ESC Guidelines for the Diagnosis and Treatment of Acute and Chronic Heart Failure” [13] before and treated in the outpatient clinic from June 2014 to December 2015. The inclusion criteria were as follows: 1. 18 to 80 years of age; 2. Echocardiography showing left ventricular systolic dysfunction (EF < 50%); 3. Not hospitalized in the past month; and 4. Standardized drug treatment regimens. Patients were excluded if they met any of the following criteria: 1. Systolic blood pressure < 90 mmHg or haemodynamic instability; 2. Second-degree type II AV block or severer; 3. HR < 55 beats/min in conscious resting state; 4. Significant abnormal liver or kidney function; 5. Malignancy; 6. Pregnant or lactating women; and 7. Dementia or mental disorder. The study design and population are detailed in Fig. 1**.**

All participants signed written informed consent forms. The study conformed to the principles outlined in the Declaration of Helsinki and was approved by relevant ethical bodies. Registration site: http://www.chictr.org.cn. Registration number: ChiCTR-ONC-14004463.

### Procedural management

A group of senior professors from The First Affiliated Hospital of Xi’an Jiaotong University formed a coaching team to conduct unified and standardized training for the clinicians of the two hospitals through centralized training and prescription instruction before patient enrolment, according to the guidelines [13] to ensure consistent treatment management principles to reduce deviations in patient treatment regimens arising from different medical units and physicians. The biomarker data generated in this prospective study were not used for treatment decisions.

On the day of enrolment, the patients were examined by uniformly trained physicians and systematically measured for body weight, blood pressure and heart rate. Heart failure symptoms were assessed using the New York Heart Association functional classification, and a 12-lead ECG was performed. Echocardiography was performed using Philips iE33 ultrasound system (Philips, Amsterdam, Netherlands) by experienced cardiologists independent of the 2 hospitals. The left ventricular ejection fraction (LVEF) value was uniformly measured by biplane Simpson rule. At last physicians recorded the treatment regimens.

### Blood sample

Blood samples were obtained by venipuncture and centrifuged at 1700 g to separate EDTA plasma and serum at once and shipped to a central laboratory with standardized procedures. Serum was collected for analysis including liver, kidney and lipids function-related tests and electrolytes (HITACHI 7180, HITACHI, Tokyo, Japan). Full blood samples were used to test the hematologic parameters (KX 21 N analyzers, Sysmex, Kobe, Japan). After these tests, all samples were stored at − 80 °C for future analysis. The serum NT-proBNP levels were detected as a batch analysis in a central laboratory by electrochemiluminescence immunoassay (Roche Diagnostics, Rotkreuz, Switzerland).

Plasma S1P was measured by liquid chromatography-tandem mass spectrometry. We used a previously described protocol with minor modifications [14]. Plasma (100 μL) was deproteinated by the addition of methanol (400 μL). The internal standard D-erythro-C17-sphingosine-1-phosphate (10 μL, 10 μmol/L, Avanti Polar Lipids Inc. Alabaster, USA) with the m/z 366.4 to 250.4 was used to correct for variations in sample preparation and instrument response. Extracts were cleared by 5810R EPPENDORF centrifugation (12,000 rpm, 10 min) (EPPENDORF, Wesseling-Berzdorf, Germany) and subjected to reverse-phase chromatography on an Agilent Eclipse XDB C-18 analytical column (2.1 mm × 150 mm, 3.5 μm) (Agilent Technologies Inc. Santa Clara, USA) at 1a flow rate of 0.3 ml/min. S1P was eluted by a ballistic gradient (30 to 85% methanol, 0.2% formic acid; % = volume%) and measured by a Shimadzu HPLC system coupled to an API 4000 tandem mass spectrometer (Applied Biosystems/MDS SCIEX, Framingham, USA). The quantification determination was performed using multiple reaction monitoring (M + H S1P parent ion). A calibration curve (0.125–5 μmol/L S1P) was generated to calculate S1P level in samples. The same sample was tested multiple times with a relative standard deviation less than 5% within 12 h, and less than 10% within 6 days when stored at room temperature.

### Assessment of outcomes

The primary endpoints were all-cause mortality and hospitalization due to aggravation of heart failure. The occurrence of patient end point events was determined through telephone follow-ups of the patients and their families, a review of hospital medical records and death certificates. All 50 all-cause deaths satisfied the definition of cardiovascular death. Therefore, no further statistical analysis was performed on cardiovascular death as secondary outcome. And we used the “average No. of hospitalization per person per year” to adjust the multiple hospitalizations and different length of follow-up. The follow-up ended on December 31, 2017 or patient death.

### Statistical analysis

The Kolmogorov-Smirnov test was used to test whether a variable conformed to a normal distribution. Normal distribution variables were expressed as the mean ± standard deviation (SD), and Student’s T test was used for comparisons between groups. Variables that did not conform to a normal distribution were expressed as the median [interquartile 1, interquartile 3], and the Mann-Whitney U test was used for inter-group comparison. Patients were grouped according to the baseline S1P level quartiles (Quartile1 (Q1), Quartile2 (Q2), Quartile3 (Q3) and Quartile4 (Q4) group). The baseline characteristics among the 4 groups were analyzed by the Kruskal-Wallis test for nonparametric variables, ANOVA for parametric variables, and the χ2 test for categorical variables. Spearman and Pearson correlation analyses were used to explore the correlations.

We analyzed S1P level as a continuous variable, fitting a restricted cubic spline function with 4 knots (located at the 5th, 35th, 65th, and 95th percentiles), and 1.10 μmol/L (The median S1P level in control group.) was chosen as the reference for all spline plots [15].

Cox proportional hazard analysis was used to determine the relationship between S1P and all-cause death. The Q2 group was selected as the reference category because it has the largely normal S1P levels and associated with the lowest risk of all-cause mortality [16]. We calculated the Meta-analysis Global Group in Chronic Heart Failure (MAGGIC) scores [17] of all patients to fit a multivariable Cox regression model. The multivariable Cox models are shown as follow. Model 1: S1P was adjusted by age and gender. Model 2: S1P was adjusted by MAGGIC score. Model 3: On the basis of Model 2, lgNTproBNP was added. The only missing data in the final model were 2 patients for NT-proBNP (< 1%), and we replaced the two missing NT-proBNP by the previous nonmissing value. As a result, the final model contained all the 210 observed patients, 50 events and no more than 5 variables which were identified to be independent predictors of all-cause death. The ability of S1P to improve death risk prediction beyond using traditional risk factors was assessed using indexes of discrimination as measured by integrated discrimination improvement (IDI) and reclassification as measured by the net reclassification index (NRI).

Survival curves were using the Kaplan-Meier method and the log-rank test was used for comparison. A 2-tailed *P* value of less than 0.05 was considered statistically significant. SPSS22.0 (SPSS, Inc., Chicago, IL, USA), STATA13.1 (StataCorp, Inc., Texas, USA) and GraphPad prism 5.0 (GraphPad Software, Inc., Cary, NC) were used for statistical analysis and graphing.

## Results

### Baseline and distribution characteristics of S1P levels

**Fig. 2:**
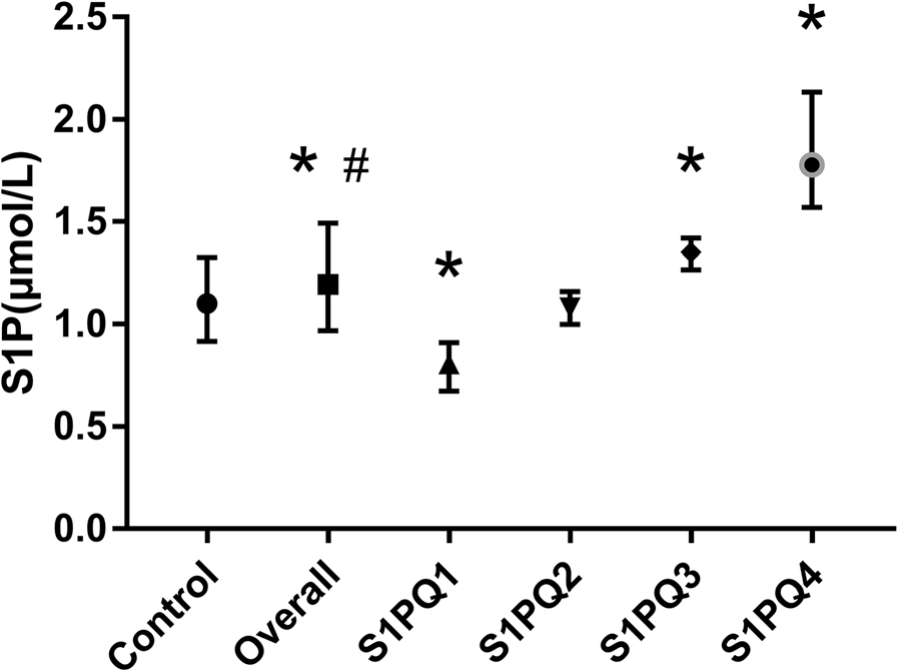
Plasma S1P levels and quartile grouping. Patients were divided into 4 groups according to the quartiles of baseline plasma S1P levels, and the plasma S1P levels of 54 healthy volunteers in the same area were used as the control group. *indicates *P*<0.05 in the Mann-Whitney U test when compared with the control group, and # indicates *P*<0.05 in the Levene test when compared with the control group. The error line is standard deviation. Abbreviations: S1P, sphingosine-1-phosphate

Among the CHF patients who completed the follow-up, 210 patients had plasma S1P test results. The patients were grouped according to the baseline plasma S1P level quartiles, and 54 healthy volunteers in the same area were selected as controls. The comparison results of S1P level are shown in Fig. 2. The overall plasma S1P of CHF patients had a higher mean value (1.269 μmol/L vs 1.122 μmol/L, *P* = 0.006) and a larger standard deviation (0.441 vs 0.316, *P* = 0.022) than those in the control group. Compared to the control group, S1PQ1 was lower, S1PQ3 and S1PQ4 were higher, and S1PQ2 showed no statistical difference.

The baseline data for the entire study population and the occurrence of outcomes in each quartile are presented in Table 1. The average age of the patients was 61.7 (SD 9.3) years, males accounted for 63.8%, LVEF was 35.76 (SD 7.57) %, NT-proBNP was 1633.5 [857.33–3123.25] pg/mL, 9.1% of patients had a history of diabetes, and 31.3% had a history of hypertension. Groups with higher S1P levels had a higher red blood cell count (*P <* 0.001), haemoglobin (*P* = 0.018), monocyte count (*P <* 0.001), and percentage of monocytes (*P <* 0.001). The neutrophil count (*P* = 0.001) and percentage of neutrophils (*P* = 0.007) showed the opposite trend. In ultrasonic cardiogram, LVEF% (*P* = 0.04) and LVEDV (*P* = 0.039) differed between the groups. Although we used the uniform principles, β-receptor blockers were used more frequently in the Q3 group, which might be related to the fewer contraindications for drug use. The usage rates of other drugs did not significantly differ between the groups.

**Table 1:** Baseline characteristics of the study population

				S1P quartiles	(*n* = 210)	Q4 > 1.490 μmol/L *n* = 52	*P*-value ^**a**^
	Control group *n* = 54	All, *n* = 210	Q1 < 0.967 μmol/L *n* = 52	Q2(0.967–1.192 μmo l/L) n = 53	Q3(1.200–1.490 μmo l/L) *n* = 53		
**Demographic characteristic**							
Age, years	56.3 ± 7.8	61.7 ± 9.32	62.23 ± 9.48	62.23 ± 7.87	60.13 ± 9.44	62.54 ± 10.41	0.559
Sex, male n (%)	20 (37.0%)	134 (63.8)	37 (71.2)	33 (62.3)	28 (52.8)	38 (73.1)	0.195
**Measurements at baseline**							
Body mass index, kg/m^2^	24.92 ± 3.38	23.1 ± 3.91	22.74 ± 3.48	22.66 ± 3.26	23.98 ± 4.45	23.02 ± 3.91	0.276
SBP, mmHg	125.89 ± 17.57	121.73 ± 21.32	122.33 ± 19.96	122.3 ± 20.99	117.92 ± 17.95	124.67 ± 26.07	0.446
DBP, mmHg	78.89 ± 9.34	76.26 ± 11.65	76.25 ± 11.69	77.06 ± 10.62	75.74 ± 11.62	75.96 ± 12.97	0.944
Heart rate, beats/min	67.0 [63.5–72.5]	74.0 [67.0–85.0]	70.5 [64.3–83.8]	73.00 [65.5–82.0]	76 [70.0–90.5]	74 [66.5–86.75]	0.127
NYHA class,							
I, n(%)	–	39 (18.6)	11 (21.2)	8 (15.1)	12 (22.6)	8 (15.4)	0.579
II, n(%)	–	120 (57.1)	27 (51.9)	32 (60.4)	26 (49.1)	35 (67.3)	
III, n(%)	–	51 (24.3)	14 (26.9)	13 (24.5)	15 (28.3)	9 (17.3)	
MAGGIC score	–	17.6 ± 5.4	18.5 ± 5.7	17.2 ± 4.1	17.2 ± 5.8	17.6 ± 5.8	0.607
eGFR,ml/min/1.73 m^2^	120.27 ± 17.94	87.29 ± 20.55	90.97 ± 19.24	84.33 ± 21.22	84.18 ± 19.89	89.98 ± 21.39	0.194
Uric acid, μmol/L	255 ± 53.36	328.1 ± 89.92	304.53 ± 100.80	327.36 ± 87.59	339.73 ± 86.53	340.33 ± 81.85	0.148
Triglyceride, mmol/L	–	1.70 ± 1.40	1.51 ± 1.12	1.74 ± 1.33	1.70 ± 0.96	1.80 ± 1.12	0.943
HDL, mmol/L	–	1.16 ± 0.33	1.13 ± 0.26	1.10 ± 0.31	1.16 ± 0.34	1.24 ± 0.37	0.341
LDL, mmol/L	–	2.39 ± 0.74	2.40 ± 0.69	2.08 ± 0.77	2.72 ± 0.67	2.34 ± 0.72	**0.003**
Erythrocyte,10^12/L	–	4.59 ± 0.91	4.40 ± 0.55	4.43 ± 0.57	4.62 ± 0.66	4.92 ± 0.91	< **0.001**
Hemoglobin, mmol/L	–	146.71 ± 22.95	142.09 ± 17.08	141.29 ± 20.20	152.23 ± 20.41	151.11 ± 30.29	**0.018**
Platelet,10^9/L	–	167.5 ± 63.0	169.7 ± 75.0	152.5 ± 55.1	179.1 ± 55.5	168.8 ± 63.5	0.182
Leukocyte,10^9/L	–	5.30 [4.50–6.20]	5.10 [4.40–6.00]	5.30 [4.75–6.75]	6.20 [5.05–7.85]	5.1 [4.23–5.60]	< **0.001**
LY10^9/L	–	1.60 [1.30–2.10]	1.50 [1.25–2.05]	1.60 [1.20–1.90]	1.77 [1.50–2.20]	1.50 [1.23–1.89]	**0.019**
LY%	–	30.71 ± 8.90	31.25 ± 9.18	29.37 ± 9.96	30.32 ± 7.66	32.04 ± 8.66	0.469
MONO, 10^9/L	–	0.30 [0.20–0.43]	0.19 [0.11–0.32]	0.31 [0.19–0.47]	0.36 [0.30–0.50]	0.32 [0.235–0.395]	< **0.001**
MONO%	–	5.70 [4.00–7.10]	3.35 [2.35–5.75]	5.85 [3.80–6.65]	6.15 [5.00–7.50]	6.6 [5.4–7.7]	< **0.001**
NEUT10^9/L	–	3.10 [2.38–3.80]	3.1 [2.50–3.80]	3.30 [2.34–4.30]	3.6 [2.8–4.8]	2.7 [2.2–3.3]	**0.001**
NEUT%	–	58.93 ± 10.56	60.94 ± 9.61	60.23 ± 10.61	59.84 ± 8.88	54.24 ± 12.06	**0.007**
**Echocardiography**							
LVEF,%	–	35.76 ± 7.57	35.05 ± 8.75	37.25 ± 6.56	33.66 ± 7.14	37.11 ± 7.30	**0.04**
LVEDD, mm	–	69.25 ± 8.86	70.29 ± 9.66	67.98 ± 8.11	69.96 ± 10.10	68.79 ± 7.35	0.518
LVESD, mm	–	56.68 ± 9.12	57.87 ± 10.02	55.15 ± 8.10	57.6 ± 10.48	56.12 ± 7.55	0.377
LVEDV, mL/m^2^	–	210.01 ± 76.12	230.46 ± 83.55	187.76 ± 65.60	213.87 ± 82.17	207.46 ± 58.74	**0.039**
LVESV, mL/m^2^	–	143.56 ± 61.05	154.98 ± 69.41	124.73 ± 51.84	147.23 ± 65.35	146.87 ± 52.98	0.07
**Biomarkers**							
S1P, μmol/L	1.100 [0.917–1.325]	1.196 [0.966–1.493]	0.806 [0.675–0.911]	1.08 [1.00–1.16]	1.350 [1.265–1.420]	1.779 [1.570–2.135]	< **0.001**
NT-proBNP, pg/mL	42.1 [35.5–56.5]	1633.5 [857.3–3123.3]	2155 [1093–3615]	1465 [716–3254]	1462 [778–2657]	1620.5 [634.5–2890.5]	0.416
**Cardiac medication, n (%)**							
ACEI/ARBs	–	185 (88.1)	45 (86.5)	46 (86.8)	50 (94.3)	44 (84.6)	0.427
Beta blocker	–	169 (80.5)	38 (73.1)	41 (77.4)	51 (96.2)	39 (75.0)	**0.009**
Diuretics	–	118 (56.2)	29 (55.8)	28 (52.8)	32 (60.4)	29 (55.8)	0.89
Aldosterone receptor antagonism	–	162 (77.1)	44 (84.6)	36 (67.9)	40 (75.5)	42 (80.8)	0.197
**Outcomes**							
All-cause mortality, n (%)	–	50 (23.8)	16 (30.8)	6 (11.3)	12 (22.6)	16 (30.8)	**0.05**
HF hospitalization, n (%)	–	121 (57.6)	32 (61.5)	27 (50.9)	33 (62.3)	29 (55.8)	0.607
All-cause mortality in the first two-year, n (%)	–	30 (14.3)	9 (17.3)	3 (5.7)	5 (9.4)	13 (25.0)	**0.023**
HF hospitalization in the first two-year, n (%)	–	100 (47.6)	28 (53.8)	20 (37.7)	24 (45.3)	28 (53.8)	0.283
Average length of follow-up, year ^**b**^	–	2.64 ± 0.86	2.57 ± 0.87	2.93 ± 0.73	2.82 ± 0.84	2.24 ± 0.84	< **0.001**
Average No. of hospitalization per person per year ^**c**^	–	0.68 [0.53–0.82]	0.79 [0.45–1.12]	0.46 [0.19–0.74]	0.44 [0.28–0.60]	0.82 [0.45–1.20]	0.307

Continuous, normally distributed variables are reported as the mean ± SD, otherwise as the median [IQ1-IQ3] or n (%). ^**a**^The baseline characteristics among the 4 quartile groups were analyzed by the Kruskal–Wallis test for nonparametric variables, ANOVA for parametric variables, and the χ2 test for categorical variables. ^**b**^Because of the lowest all-cause mortality rate, more patients in the S1PQ2 group could reach the end of the follow-up (December 31, 2017). So that S1PQ2 group had the longest average length of follow-up. ^**c**^“Average No. of hospitalization per person per year” was used to adjust the multiple hospitalizations and different length of follow-up. Abbreviations: ACEI, angiotensin-converting enzyme inhibitor; ARB, angiotensin II receptor blocker; DBP, Diastolic blood pressure; eGFR, estimated glomerular filtration rate; NYHA, New York Heart Association; HDL, high-density lipoprotein; LDL, low density lipoprotein; LY, leukomonocyte; LV, left ventricle; LVEF, left ventricular ejection fraction; LVEDD, left ventricular end-diastolic dimension; LVESD, left ventricular end-systolic dimension; LVEDV, left ventricular end-diastolic volume; LVESV, left ventricular end-systolic volume; MONO, monocyte; NT-proBNP, N-terminal pro B-type natriuretic peptide; NEUT, neutrophil; S1P, sphingosine-1-phosphate; SBP, Systolic blood pressure;

The correlation analysis showed that the plasma S1P level was positively correlated with the red blood cell count (r = 0.312, *P <* 0.001), haemoglobin (r = 0.189, *P* = 0.006), monocyte count (r = 0.287, *P <* 0.001) and monocyte percentage (r = 0.412, *P <* 0.001), but it was negatively correlated with the percentage of neutrophils (r = − 0.263, *P <* 0.001).

### Follow-up

After an average follow-up period of 31.7 (SD 10.3) months (range: 2–43 months), 50 all-cause deaths occurred in the 210 patients, and 121 patients were re-hospitalized due to exacerbation of heart failure (Table 1). All 50 all-cause deaths satisfied the definition of cardiovascular death. Therefore, no further statistical analysis was performed on cardiovascular death as secondary outcomes. The Q2 group had the lowest all-cause mortality and proportion (*n* = 6, 11.3%), while Q3 (*n* = 12, 22.6%), Q4 (*n* = 16, 30.8%) and Q1 group (*n* = 16, 30.8%) showed increased all-cause mortality. The similar trend could be found in all-cause mortality and hospitalization rates during the first 2 years of follow-up and the average No. of hospitalizations per person per year. However, there was no significant difference in the HF re-hospitalization rate between the groups.

### Prognostic value of baseline S1P levels

**Fig. 3:**
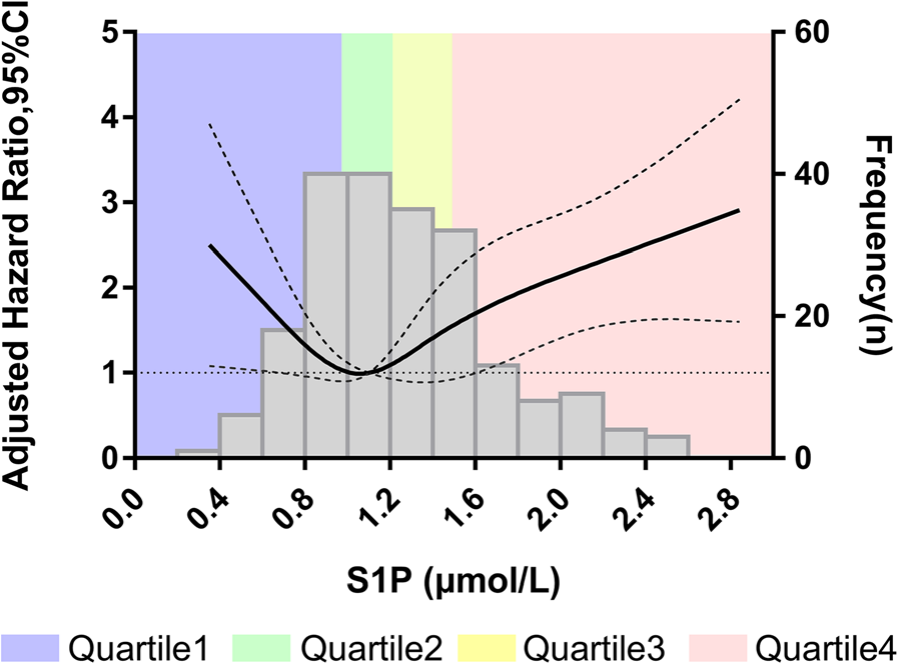
Association between S1P levels and the adjusted hazard ratio for all-cause death. Histograms stand for frequency of patients at different S1P concentrations (The right-hand y-axis). The solid curve gives expected of adjusted HR based on restricted cubic spline analysis (The left-hand y-axis). The dashed curves represent the 95% confidence intervals for expected of adjusted HR (The left-hand y-axis). Model adjusted on MAGGIC score and lgNT-proBNP

Based on univariable Cox regression with restricted cubic spline analysis, a non-linear and U-shaped association between S1P levels and risk of all-cause death was observed (Fig. S1). And the trend still existed in adjusted HR spline (Fig. 3). The inflection point of U-shaped spline was 1.06 μmol/L. Patients with a S1P level of 1.06 μmol/L had the lowest risk of all-cause death, which was consistent with the results in Fig. 2, where the S1P level of the Q2 group and the control group did not significantly differ. We divided patients into two groups according to the inflection point. In the group with S1P level ≥ 1.06 μmol/L, patients with lower S1P had significantly better survival (Adjusted HR = 2.368 [1.006–5.572], *P* = 0.048). In patients with S1P level < 1.06 μmol/L, a greater decrease in S1P level was associated with increased risk of all-cause death (Adjusted HR = 0.041[0.002–0.808], *P* = 0.036). The U-shaped association was robust in the multivariate analyses (Table S1).

Then we analyzed S1P level as a category variable according to the quartiles. The Cox proportional hazard analysis results are shown in Table 2. The patient plasma S1P level belonging to the Q1 group, the patient plasma S1P level belonging to the Q4 group, lgNT-proBNP, DBP, uric acid, haemoglobin, LVEF, LVEDD, LVESD, LVEDV and LVESV were found to be univariate predictors of all-cause death. Univariable analysis revealed that low S1P levels (Q1 group: S1P < 0.967 μmol/L; HR: 3.271 (1.277–8.381), *P* = 0.014) and high S1P levels (Q4 group: S1P > 1.490 μmol/L; HR: 3.870 (1.504–9.960), *P* = 0.005) were both associated with the high risk of all-cause death. Multivariable analysis showed that patient plasma S1P level belonging to the Q4 group (adjusted HR 3.418; 95%CI 1.320–8.849; *P* = 0.011) were independent predictors of all-cause mortality, while the prognostic value of patient plasma S1P level belonging to the Q1 group (adjusted HR: 2.525; 95%CI: 0.972–6.563; *P* = 0.057) subsided after additional adjustment for MAGGIC score and lgNT-proBNP.

**Table 2:** Cox regression analysis for S1P and all-cause death

Model	HR ratio(95%CI) for S1PQ1	*P*-value	HR ratio(95%CI) for S1PQ4	*P*-value
S1P Crude HR	3.271 (1.277–8.381)	**0.014**	3.870 (1.504–9.960)	**0.005**
**Model 1** S1P + age + gender	3.267 (1.274–8.378)	**0.014**	3.885 (1.508–10.008)	**0.005**
Adjustments for predictors of all-cause death in CHF				
S1P + lgNT-proBNP	2.634 (1.019–6.808)	**0.046**	3.504 (1.362–9.017)	**0.009**
S1P + DBP	3.197 (1.248–8.188)	**0.015**	3.984 (1.546–10.265)	**0.004**
S1P + LVEF%	2.785 (1.072–7.235)	**0.036**	3.742 (1.453–9.637)	**0.006**
S1P + LVEDD	2.922 (1.131–7.546)	**0.027**	3.723 (1.447–9.578)	**0.006**
S1P + LVESD	2.850 (1.101–7.378)	**0.031**	3.701 (1.438–9.526)	**0.007**
S1P + LVEDV	2.689 (1.032–7.007)	**0.043**	3.504 (1.360–9.030)	**0.009**
S1P + LVESV	2.759 (1.063–7.164)	**0.037**	3.415 (1.321–8.825)	**0.011**
Adjustments for independent predictors of all-cause death in CHF				
S1P + lgNT-proBNP+LVEDV	2.043 (0.771–5.413)	0.115	3.144 (1.220–8.101)	**0.018**
S1P + lgNT-proBNP+DBP	2.420 (0.932–6.281)	0.069	3.342 (1.291–8.655)	**0.013**
S1P + LVEDV+DBP	2.574 (0.981–6.750)	0.055	3.736 (1.449–9.630)	**0.006**
**Model 2** S1P + MAGGIC score	2.838 (1.097–7.344)	**0.032**	3.878 (1.505–9.991)	**0.005**
**Model 3** S1P + MAGGIC score + lgNT-proBNP	2.525 (0.972–6.563)	0.057	3.418 (1.320–8.849)	**0.011**

Abbreviations: *HR* hazard ratio; *MAGGIC score* Meta-analysis Global Group in Chronic Heart Failure score; *NT-proBNP* N-terminal pro B-type natriuretic peptide; *S1P* sphingosine-1-phosphate

According to the S1P optimal thresholds obtained by the restricted cubic spline, patients can be divided into high risk group and low risk group. Adding S1P classification to the model with MAGGIC score and lgNT-proBNP, we found a 39.8% (Z = 2.425, *P* = 0.015) improvement in the NRI (Table S2) and a 0.027 (Z = 2.226, *P* = 0.026) improvement in the IDI.

**Fig. 4:**
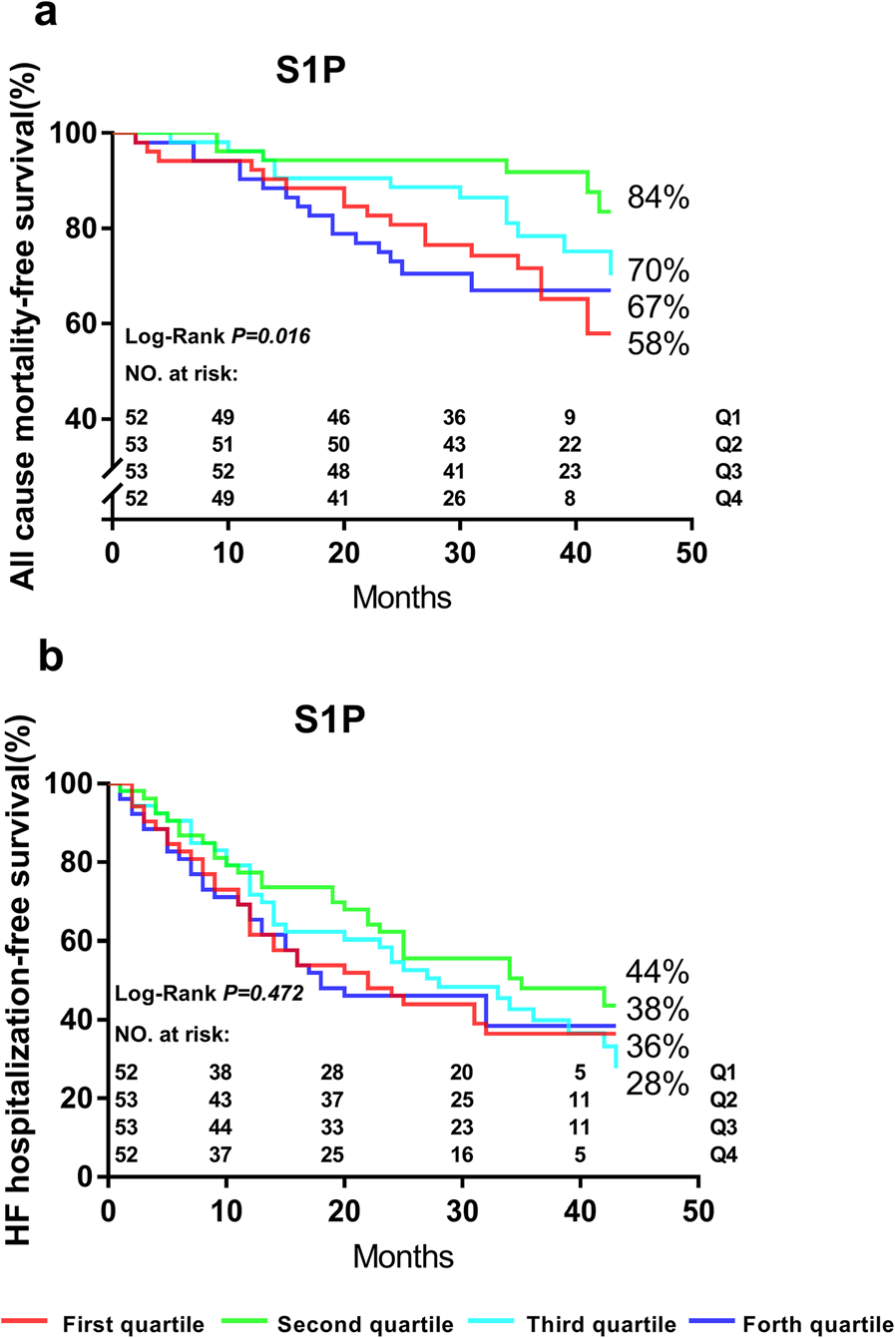
Kaplan-Meir curves of all-cause mortality (**a**) and heart failure hospitalization (**b**). Abbreviations: S1P, sphingosine-1-phosphate

The survival curves in Fig. 4 showed that plasma S1P levels in the lowest quartile (S1P < 0.967 μmol/L) and highest quartile (S1P > 1.490 μmol/L) were at a higher risk of death. But there was no significant difference in the re-hospitalization rate due to aggravated heart failure between the groups.

## Discussion

The main finding of this study was that plasma S1P in patients with heart failure was associated with all-cause mortality, which showed a U-shaped correlation. And we also described the overall distribution of S1P levels in heart failure patients and first provided CHF patients normal (low risk) S1P thresholds for reference.

### U-shaped relationship bridges the gap from basic study to clinic patients

In this study, we found that S1P had different distribution characteristics in the normal population and CHF patients. Abnormally increased (Q3, Q4 group), decreased (Q1 group) and roughly normal S1P levels (Q2 group) coexisted in CHF patients. Because abnormal decrease had less amplitude and room than increase, the CHF group showed a higher mean value (1.269 μmol/L vs 1.122 μmol/L, *P* = 0.006) and standard deviation (0.441 vs 0.316, *P* = 0.022) of S1P level than the control group. Combined with the restricted cubic spline results, where patients with roughly normal S1P levels (Q2 group) had the lowest mortality rate, these results suggest that the S1P level is associated with all-cause mortality in a U-shaped relationship, indicating that there may be a normal value range for S1P levels in the middle of the entire distribution rather than on either side.

In previous animal model studies, different results were reported for the S1P/S1PR axis function.

Some of the studies have found that S1P and S1PR expression decreased after heart injury which can be improved by upregulating S1P level or S1PR activity. In the model of post-ischemic HF, Cannavo A et al. demonstrated that either cardiac or circulating levels of S1P are reduced compared to non-ischemic control [6]. S1PR1 receptor expression decreased after myocardial infarction, and chronic S1P treatment could arrest post-MI HF progression [5]. Meanwhile, S1P lyase activation in the myocardium following ischemia leads to reduced S1P levels and that knockout mice for this enzyme exhibit higher S1P levels and smaller infarct size [18]. In addition to the ischemia model, Yan H et al. [19] demonstrated that exogenous S1P improved rat cardiac hypertrophy induced in the transverse aortic constriction model. Similar conclusions were reached in studies of FTY720 (A kind of high-affinity agonist of S1PR1 and S1PR3–5). Hofmann U et al. [20] have tested the hypothesis that FTY720 can improve functional recovery when applied with reperfusion after myocardial ischaemia. Yeh CC et al. [10] showed that S1P levels decreased after myocardial infarction in rats and that administration of FTY720 reduced apoptosis and ventricular remodelling after infarction. Santos-Gallego CG et al. [21] found that S1PR activation with FTY720 during acute MI reduced infarct size via the reperfusion injury salvage kinase and survivor activating factor enhancement pathways, improved systolic LV function, and mitigated post-MI LV remodeling.

Other studies have found that S1P levels increased after heart injury. Zhang F et al. [22] showed that plasma S1P levels, Sphk1 and S1PR1 activities increased after myocardial infarction in rats and that downregulation of S1P function by an inhibitor could protect the heart. In another mouse model study [23], 10-week-old C57Bl6 mice underwent experimental myocardial infarction. At 1 to 12 weeks postoperative, S1P was increased as early as 1 week after MI and remained elevated. Empinado HM et al. [24] made similar findings. In their research myocardial infarction was used to induce CHF in rats. Heart failure increased diaphragm S1P level. They found that the levels of S1P in CHF group were increased twofold than control group.

These studies used different animal models, observed different time points during the disease process, and identified different S1P change directions. But fortunately, the similarity of these studies is that either an increase or a decrease of S1P levels in heart failure animal models is harmful, and regulation of abnormal S1P level towards the control level is beneficial for heart protection. We identified both elevated and decreased S1P levels in clinical patients and a U-shaped association with all-cause deaths, allowing different directions of previous animal model studies to be simultaneously reproduced at the clinical level.

In summary, before this study, there was insufficient information to suggest that either upregulate or downregulate S1P signaling will be effective in the setting of heart failure [11]. For the first time, our study showed a U-shaped relationship between the S1P levels and all-cause death, and provided CHF patients normal (low risk) S1P thresholds for reference. Through restoring abnormal levels to a normal range instead of simply up-regulation or down-regulation, S1P has the potential to be a therapeutic target for reducing the risk of death in patients with heart failure in the future.

### U-shaped relationship bridges the gap between different mechanisms

At present, excessive activation of the neurohumoral endocrine system (Sympathetic nervous system and Renin-angiotensin system) and its consequent progressive ventricular remodeling are the most recognized mechanisms leading to the development of heart failure. Although an increasing body of experimental evidence supports the notion that normal S1P/S1PR system protects the heart, there is insufficient evidence to support that abnormal S1P is directly involved in the development of heart failure. Our study has the inherent limitation of observational research, that is, the causal relationship between abnormal S1P level and the development of heart failure cannot be explained from the mechanism, but it can provide hypothesis for the direction of future research.

In this study, the prognostic value of decreased plasma S1P levels for all-cause mortality diminished after correction with NT-proBNP and LVEDV. The LVEDV is an independent risk factor for all-cause death and also presents the degree of left ventricular remodeling in heart failure. The S1PQ1 group had higher LVEDV than S1PQ2 group (230.46 (SD 83.55) vs 187.76 (SD 65.60), *P* = 0.007). And Cannavo A et al. [5] showed a mutual regulation mechanism between S1P/S1PR function and β-adrenergic receptor function. These results suggest that an abnormal decline of plasma S1P may be associated with heavier ventricular remodelling. Furthermore, Gazit SL et al. [25] showed that S1P deficiency may aggravate vasoplegia, arguing a vital role for S1P in maintaining vascular resistance during recovery from circulatory shock. The results may explain the relationship between the increased all-cause mortality and low S1P levels in some patients.

The prognostic value of an abnormal elevation of sphingosine-1-phosphate (S1P) for death is completely independent of the previous high-risk factors and the prediction indexes associated with heart failure. After adjusting for variables associated with S1P and risk factors for all-cause death in heart failure, the prognostic value of S1P was not reduced, suggesting the presence of a new mechanism for the occurrence and development of heart failure that is independent of previously identified conventional risk factors. As an endogenous sphingolipid molecule with important biological activity, S1P has important immunomodulatory functions [26, 27] that S1P/S1PR abnormally changes in autoimmune diseases such as rheumatoid arthritis [28] and in severe infectious diseases such as sepsis [14]. The excessive activation of S1P/S1PR can also lead to the occurrence of immune disorders and inflammation [29], which is also one mechanism of heart failure. The cause of S1P changes in heart failure patients, the mechanism by which excessive activation of the S1P/S1PR axis leads to increased all-cause mortality in these patients, and the relationship between the S1P/S1PR axis and immune disorders await further research for confirmation.

We hypothesize that during the occurrence and development of heart failure, the two different directions of abnormal changes in plasma S1P levels are linked to the two different mechanisms of ventricular remodelling and immune disorders, separately or simultaneously. S1P may have the potential to act as the “bridge molecule” that links the neurohumoral endocrine system and the immune system, which can be investigated in future studies on the pathophysiological process of heart failure occurrence and development.

### Limitations

There are several limitations related to our study. Because out-of-hospital CHF patients were enrolled in this study, some patients did not undergo coronary angiography, so in these cases the patients were not classified according to the aetiology of heart failure. As a result, whether different causes could affect the direction of plasma S1P could not be analysed in depth. Furthermore, we do not know the variance of S1P value if it is measured at different time points in the same patient at present. Although the all-cause mortality during the whole follow-up period and in the first two-year showed the same trend, we still wonder whether it is more appropriate to use the sequential value of S1P to predict the prognosis more than 2 years after the enrolment. Moreover, only patients with heart failure in 2 hospitals were enrolled in this study. Due to these limitations, we consider our study mainly as a hypothesis generator. Future large-scale, multicenter clinical research will effectively improve the reliability of these findings.

## Conclusions

In patients with chronic systolic heart failure, plasma S1P levels are related to the long-term all-cause mortality with a U-shaped correlation. Through restoring abnormal levels to a normal range instead of simply up regulation or down regulation, S1P may have the potential to be a therapeutic target for reducing the risk of death in patients with heart failure in the future.

## Supplementary information

**Additional file 1. Table S1.** Univariate and multivariate Cox analyses for S1P in two subgroups. **Table S2.** Net reclassification of death and not death with adding S1P to Risk Factors. **Figure S1.** Association between S1P levels and the hazard ratio for all-cause death.

## Data Availability

The datasets generated during and/or analysed during the current study are available from the corresponding author on reasonable request.

## Abbreviations

AV block: Atrioventricular block
ACEI: Angiotensin-converting enzyme inhibitor
ARB: Angiotensin II receptor blocker
CHF: Chronic heart failure
DBP: Diastolic blood pressure
ECG: Electrocardiograph
EDTA: Ethylene diamine tetraacetic acid
EF: Ejection fraction
eGFR: Estimated glomerular filtration rate
HDL: High-density lipoprotein
HR: Hazard ratio
LDL: Low density lipoprotein
LV: Left ventricle
LVEF: Left ventricular ejection fraction
LVEDD: Left ventricular end-diastolic dimension
LVESD: Left ventricular end-systolic dimension
LVEDV: Left ventricular end-diastolic volume
LVESV: Left ventricular end-systolic volume
LY: Leukomonocyte
MONO: Monocyte
NEUT: Neutrophil
NT-proBNP: N-terminal pro B-type natriuretic peptide
NYHA: New York Heart Association
S1P: Sphingosine-1-phosphate
S1PR: Sphingosine-1-phosphate receptor
SBP: Systolic blood pressure
SD: Standard deviation
