# Supplementary material for: U-shaped association between plasma sphingosine-1-phosphate levels and mortality in patients with chronic systolic heart failure: a prospective cohort study
Source: Lipids Health Dis. 2020 Jun 4;19:125. doi: 10.1186/s12944-020-01262-2 (PMC7273664; doi:10.1186/s12944-020-01262-2)
Supplement: Supplementary file 1 — Additional file 1. Table S1. Univariate and multivariate Cox analyses for S1P in two subgroups. Table S2. Net reclassification of death and not death with adding S1P to Risk Factors. Figure S1. Association between S1P levels and the hazard ratio for all-cause death. [file 12944_2020_1262_MOESM1_ESM.docx]

Table S1 Univariate and multivariate Cox analyses for S1P in two subgroups

|  | Crude HR(95%CI) | *P*-value | Adjusted HR (95%CI)**^a^** | *P*-value |
| --- | --- | --- | --- | --- |
| Patients with S1P<1.06μmol/L | 0.059(0.004-0.788) | **0.032** | 0.041(0.002-0.808) | **0.036** |
| Patients with S1P≥1.06μmol/L | 2.441(1.060-5.622) | **0.036** | 2.368(1.006-5.572) | **0.048** |

S1P was analyzed as a continuous variable. **^a^**S1P was adjusted by MAGGIC score and lgNT-proBNP. Abbreviations: HR, Hazard ratio, S1P, sphingosine-1-phosphate.

Table S2 Net reclassification of death and not death with adding S1P to Risk Factors

| Model | Reclassified Up (%) | Reclassified Down (%) | NRI |
| --- | --- | --- | --- |
| S1P(cut offs:0.68-1.62μmol/L)+MAGGIC score + lgNTproBNP | | |  |
| Death | 32.65 | 67.35 | -34.7 |
| Not death | 19.59 | 80.41 | 60.82 |
| Total |  |  | 26.12(10.64-42.60) |
| S1P(cut offs:0.91-1.49μmol/L)+MAGGIC score + lgNTproBNP | | |  |
| Death | 57.1 | 42.9 | 14.2 |
| Not death | 37.2 | 62.8 | 25.6 |
| Total |  |  | 39.8(23.5-56.3) |

Net reclassification index (NRI) (95% CI) calculated for the addition of S1P to model: MAGGIC score + lgNTproBNP. Reclassification separated by death or not during follow-up.

**Figure S1**

**
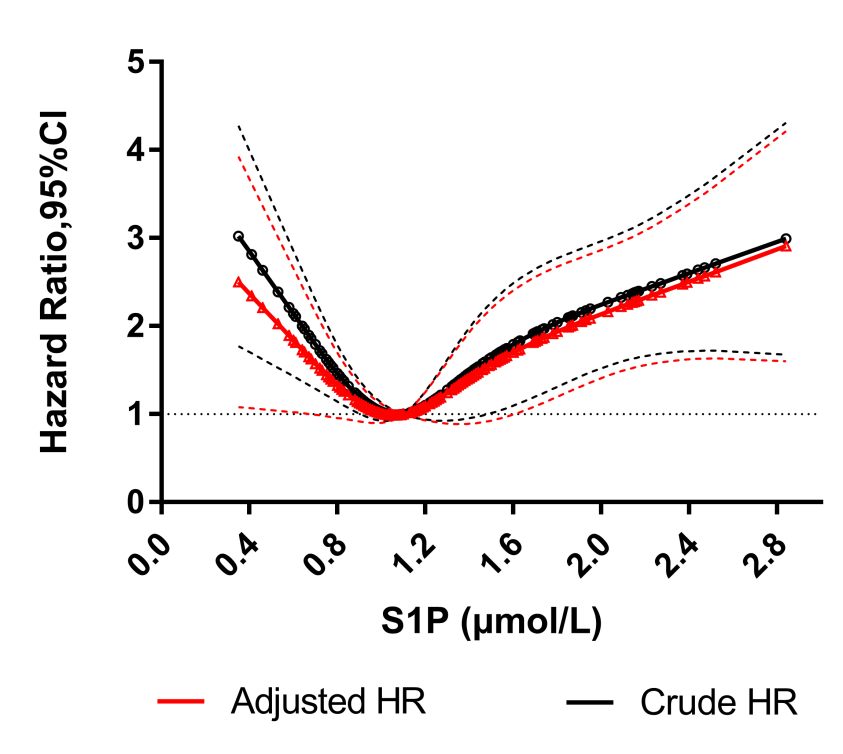
**

**Figure legend**

**Figure S1 Association between S1P levels and the hazard ratio for all-cause death. The solid curve gives expected of HR based on restricted cubic spline analysis. The dashed curves represent the 95% confidence intervals for expected of HR. The circles and triangles give HR values which were used to fit the restricted cubic spline. Model adjusted on MAGGIC score and lgNT-proBNP.**
